# Rheo-NMR velocimetry of nanocrystalline cellulose suspensions

**DOI:** 10.1515/arh-2024-0026

**Published:** 2024-01

**Authors:** Maribelle A. Stanley, Jayesha S. Jayaratne, Sarah L. Codd, Dilpreet S. Bajwa, James N. Wilking, Joseph D. Seymour

**Affiliations:** Chemical and Biological Engineering Department, Montana State University, Bozeman, MT, United States of America; Chemical and Biological Engineering Department, Montana State University, Bozeman, MT, United States of America; Mechanical and Industrial Engineering Department, Montana State University, Bozeman, MT, United States of America; Mechanical and Industrial Engineering Department, Montana State University, Bozeman, MT, United States of America; Chemical and Biological Engineering Department, Montana State University, Bozeman, MT, United States of America; Department of Physiology and Biomedical Engineering, Mayo Clinic, Rochester, MN, United States of America; Chemical and Biological Engineering Department, Montana State University, Bozeman, MT, United States of America

**Keywords:** nanocrystalline cellulose, complex fluids, colloidal suspensions, shear-banding, velocity fluctuations

## Abstract

The velocity data presented demonstrate the complicated flow behavior of nanocrystalline cellulose (NCC) suspensions even when standard rheometry shows only subtle effects. Rheo-nuclear magnetic resonance (NMR) velocimetry with spatial and temporal resolution indicates that NCC suspensions undergo varying flow behavior, which correlates with bulk rheology and includes wall-slip, shear banding, and yielding. Large-velocity fluctuations in a chiral nematic liquid crystal-phase suspension (5% w/v) indicate particle director orientation tumbling and flow. The results provide details of the mesoscale velocity distributions in space and time, which can be used to inform the interpretation of rheology data, as well as processing flow conditions to control NCC suspension microstructure and impact properties of composite and other materials.

## 1 Introduction

Cellulose is the main component of plant cell walls [1]. It is a biological polymer of glucose monomers [2] that has been heralded as the most abundant renewable compound [3]. Numerous processes seek to utilize it as a replacement for traditional feedstocks [4–6]. A relatively new process for this purpose involves bundling cellulose chains and separating them into nanoparticles or nanofibers called *nanocrystalline cellulose (NCC)* [7]. Materials formed with NCC have been regarded as “futuristic” owing to NCC’s impressive chemical and physical properties. Such properties include stiffness and strength, chemical inertness, tunable mechanics, optical birefringence, and modifiable surface chemistry, among others [7]. These advantages enable a wide variety of applications, from capacitors and food products to fibers, cement, and biomedical tissues [7–12], and a growing research interest to inform such pursuits.

To fully maximize the industrial relevance of NCCs, their flow mechanics must be understood to optimize processing conditions. NCCs form colloidal aqueous suspensions that exhibit a wide range of complex rheological behaviors [13]. This behavior depends on particle concentration, salt concentration, sonication energy, and applied shear stress [14,15]. Concentration dependence prompts changes from shear-thinning viscoelastic to liquid crystal three-region and shear-thinning polymeric gel behavior [14–16]. This broad material response in concentration-dependent behavior results from differences in phase microstructure. NCC particles are rod-like of aspect ratio $\frac{L}{d}\sim 10$ with sulfate half-ester groups that generate electrostatic repulsion [17]. The interaction free energy is a combination of geometric excluded volume and electrostatic effects [17,18]. The electrostatic repulsion generates an equilibrium-phase isotropic-nematic system at a lower critical concentration than pure geometric kinetic arrest [17,18]. As the particle concentration increases above the critical concentration, the system enters a liquid crystal chiral nematic phase and then typically isotropic gel phase, which can, however, maintain the nematic structure [17]. Sonication disperses aggregates, while shearing can disperse, break apart, and re-assemble aggregates [19,20]. Such varied behavior is a current limit to the use of NCC’s capabilities on an industrial scale.

Like many complex fluids, bulk rheological measurements provide a challenge because of microscale heterogeneity unresolvable with traditional torque-based rheological measurements. Previous studies have sought to overcome these challenges in NCCs by complementing bulk rheological measurements with optical and spectroscopic techniques [13,21]. Nuclear magnetic resonance (NMR) velocimetry is particularly suited to this challenge because of its ability to resolve heterogeneous flow behavior down to the micron scale, and pinpoint flow dynamics occurring at multiple length and time scales [22–30]. Rheo-NMR velocimetry couples the ability to shear fluids at controlled shear rates with direct observations of the spatial velocity occurring in real time [22,23,28–30]. To date, Rheo-NMR studies have focused on hydroxypropyl cellulose, a separate and somewhat less versatile cellulose liquid crystal derivative, with mainly deuterated spectral studies as opposed to NMR velocity imaging [21,31,32]. The authors are not aware of any Rheo-NMR velocimetry studies that focus specifically on NCC suspensions as pointed out in the review from Kadar and co-workers [13].

In this work, Rheo-NMR velocimetry was used to obtain transient and steady-state 1D velocity profiles of NCC suspensions in the fluid gap of a home-built, NMR-compatible concentric cylinder shear cell [29]. From these velocity distributions, the flow evolution is observed at several shear rates for a range of concentrations. Traditional bulk rheology measurement with strain-controlled measurements for the same samples complements the results. The data demonstrate four distinct regimes of flow behavior as a function of particle concentration that offer a more detailed picture of the material response to shear of NCC suspensions.

## 2 Materials and methods

### 2.1 Sample preparation

The NCC systems investigated in this work are a series of aqueous suspensions ranging from 3 to 12% w/v. Cellulose nanocrystals in the form of nanorods with dimensions 10–15 nm in width and 80–100 nm in length were obtained from the U.S. Department of Agriculture, Forest Products Laboratory (Madison, WI) [33]. The samples were prepared by weighing the cellulose nanocrystals as a freeze-dried powder and then adding the appropriate amount of reverse osmosis/deionized water with stirring. The resulting suspensions were homogenized by applying ultrasound energy from a 2510 Branson sonication ice water bath (130 W power output, 40 kHz frequency, 2.81 L bath volume); ice water (~12°C) was used in the bath to prevent the degradation of the NCC particles due to sensitive surface charges, which may cause de-esterification of sulfate groups [14,34,35]. Samples were sonicated and periodically vortexed until visually homogeneous, such that the total sonication energy for each sample was ~300 J/g of NCC, where <1,000 J/g is found to be sufficient to break aggregates and disperse the nanorods [14]. All suspensions were stored in a refrigerator (3°C) before and after measurements.

### 2.2 Rheometry

**Figure 1:**
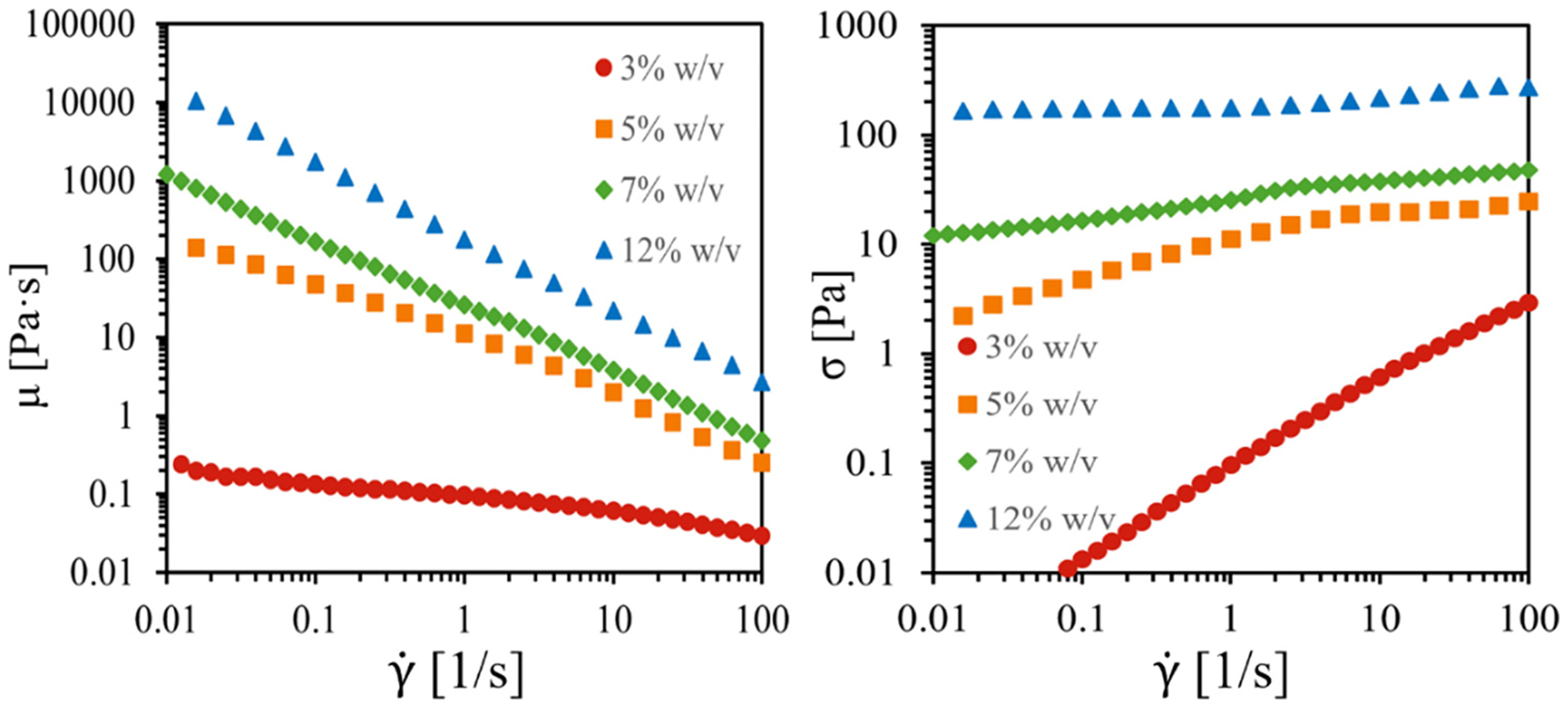
Left: viscosity vs. applied shear rate from bulk rheometry experiments on NCC suspensions, for a 3% w/v sample, which demonstrates slightly nonlinear shear-thinning behavior; a 5% w/v sample, which demonstrates a higher viscosity at low shear rates, and slight deviation from a linear decrease as shear rate is increased; a 7% w/v sample, which shows a much higher viscosity at low shear rates and a linear decrease; and a 12% w/v sample, which also shows a much higher viscosity and a linear decrease. Right: shear stress vs. applied shear rate from bulk rheometry experiments on NCC suspensions, for a 3% w/v sample demonstrating mostly Newtonian behavior and slight shear thinning; a 5% w/v sample, which exhibits the stress plateau that characterizes shear banding fluids, extending from γ=6.3 to 40/s; a 7% w/v sample, which shows a slight stress plateau and shear thinning; and a 12% w/v sample, showing slight shear thinning. The data were measured at 25°C with a 60 mm, 2° cone-and-plate for 3% w/v suspensions, and a 20 mm parallel plate geometry for 5, 7, and 12% w/v suspensions.

Linear rheological measurements were performed with an AR-G2 rheometer (TA Instruments) equipped with either a 60 mm diameter and 2° angle cone-and-plate geometry for 3% w/v suspensions or a 20 mm diameter parallel plate for 5, 7, and 12% w/v suspensions. All experiments were run at 25°C with a controlled strain rate. The viscosity and shear stress measurements (Figure 1) are in agreement with reported data for NCC suspensions with no salt and sonication levels of 300 J/g, relative to non-sonicated and 1,000 J/g sonicated samples [14].

### 2.3 Rheo-NMR velocimetry

**Figure 2:**
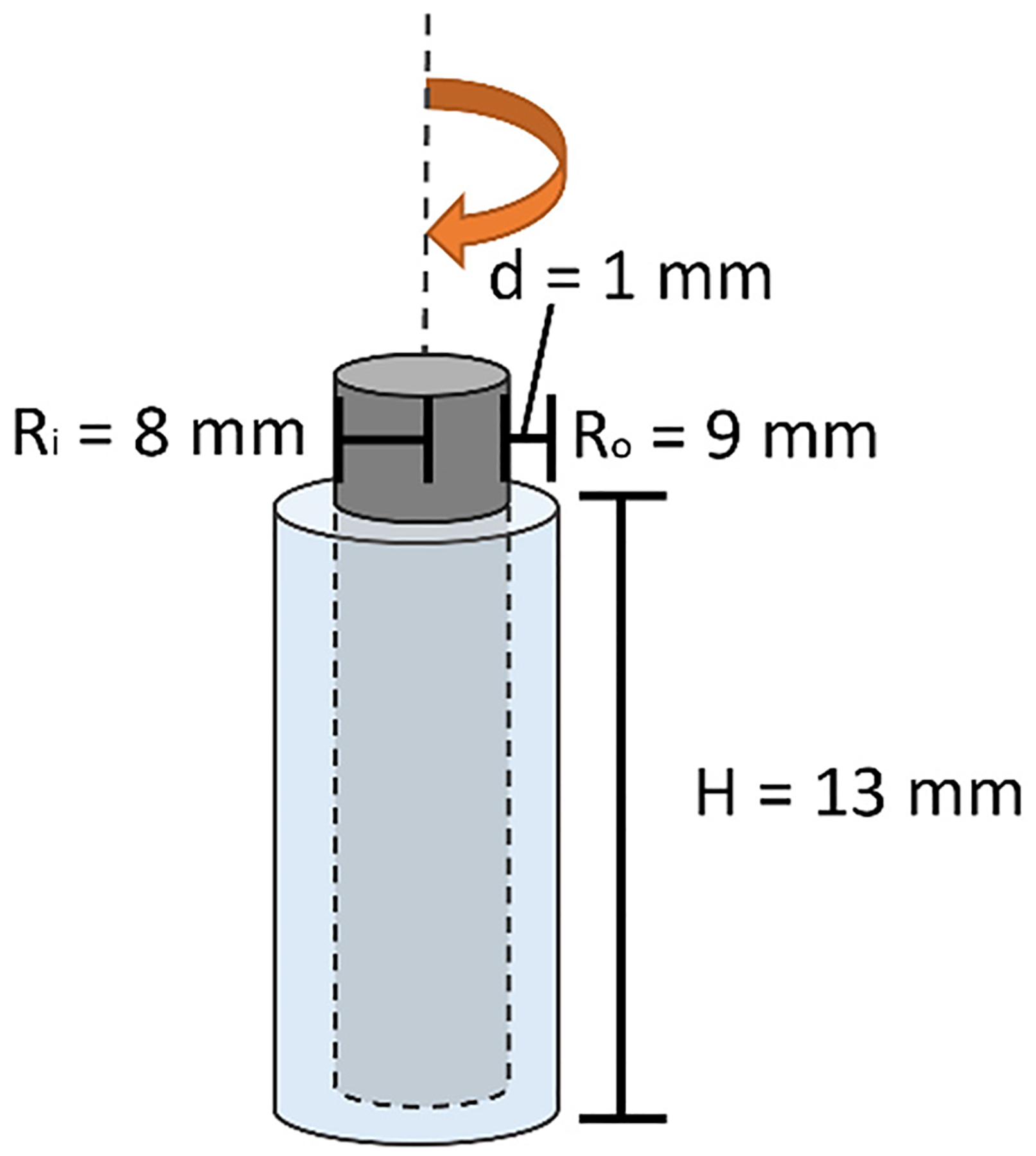
Schematic that depicts the Rheo-NMR sample Couette cell dimensions.

Velocimetry experiments were performed with a portable, home-built Rheo-NMR experimental system and methodology with well-established fluid flow measurement capabilities across a variety of length and time scales [22,36–38]. The system includes an NMR-compatible, motor-powered driveshaft, which rotates the inner rotor of a Couette shear cell, depicted in Figure 2. The shear cell has a 9 mm outer radius glass cylinder stator and 8 mm inner radius PEEK rotor. The dimensions approach a narrow gap system with fluid gap width $\left(d=R_{o}-R_{i}\right)$ of 1 mm, a radius ratio $\left(\kappa =\frac{R_{i}}{R_{o}}\right)$ of 0.89, and a q value $\left(q=\frac{d}{R_{i}}\right)$ of 0.143, so stress inhomogeneity is small but nonzero. Thus, as the shear rate inhomogeneity is also small but nonzero, the shear rate, $\dot{\gamma }$, refers to an effective shear rate. Samples were loaded into the shear cell and the 7 and 12% w/v samples were put under vacuum for 30 min to eliminate air bubbles. All samples were pre-sheared to remove air bubbles and provide a uniform microstructure initialization prior to measurements, using an applied shear rate of $\dot{\gamma }=4.6/s$ for 30 min to 1 h. The samples were allowed 3 h to relax before measurements were taken. Shear rates of $\dot{\gamma }=0.9,2.8$, and 4.6/s were applied with a calibration fluid silicone oil (10,000 cSt) used to measure the actual velocity at the rotor to experimentally determine $\dot{\gamma }$ for each motor set point.

**Figure 3:**
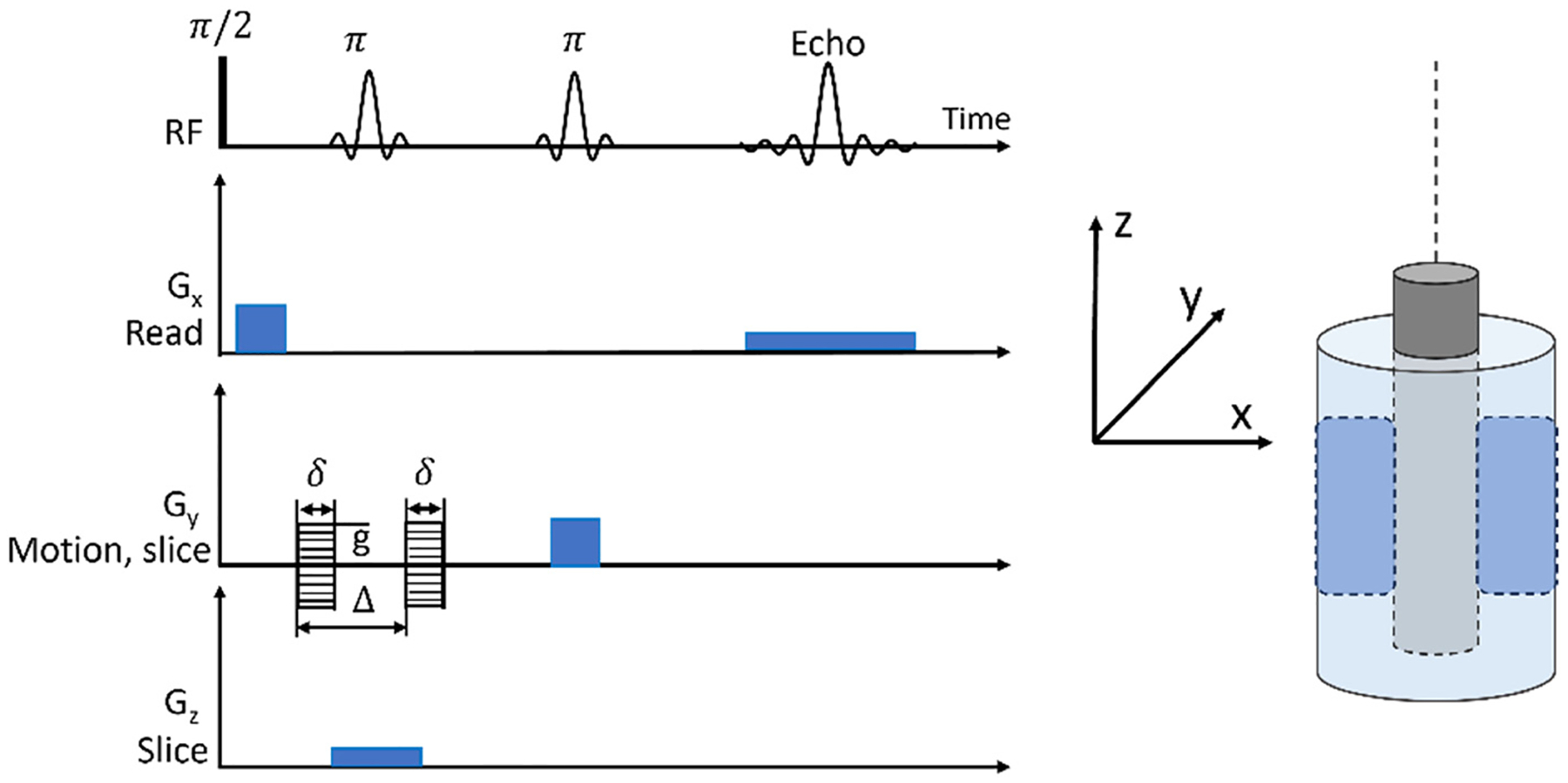
The PGSE pulse sequence with double slice selection used to acquire 1D velocity profiles and an adjacent depiction of the imaged region of the Couette flow cell. Data were acquired from a 10 mm slice in the vorticity direction (z-axis) and a 1 mm thick slice along the velocity direction (y-axis) as shown in the shaded section of the Couette schematic. The 1D profile was acquired across the fluid gap in the read gradient direction (x-axis) with a spatial resolution of 58.6 μm/pixel.

NMR measurements were taken with a Bruker AVANCE 300 MHz spectrometer and 7 T superconducting super wide bore magnet equipped with a 25 mm ^1^H birdcage resonator coil and a Micro-2.5 gradient system (providing a maximum gradient of 1.5 T/m at 60 A). Bruker TopSpin 3.5pl7 software was used to acquire data with the spectrometer, while MATLAB (MathWorks, Inc., Natick, MA) and Prospa software (Magritek, Wellington, NZ) were used to analyze the data. A pulsed gradient spin echo (PGSE) pulse sequence (Figure 3) was used to acquire 1D velocity profiles of the liquid phase from the protons of the fluid molecules. The PGSE pulse sequence encodes for magnetization phase, ϕ, where $\phi =\gamma v\int g\left(t^{\prime }\right)t^{\prime }dt^{\prime }=\gamma g\delta \Delta \cdot v$. Here, γ refers to the magnetogyric ratio (a material property), g refers to the gradient amplitude, δ refers to the gradient pulse duration, and Δ refers to the displacement observation time. The PGSE pulse sequence was employed with double slice selection [23,29]. The slices used were a 1 mm thick slice in the flow direction (y) to eliminate curvature and provide $v_{\theta }(r)$ azimuthal radius-dependent velocity directly over a 10 mm slice in the vertical direction (z) to provide and average more ^1^H spins over the vorticity direction of the flow and increase signal to noise for two average 8 s acquisitions. Two gradient steps of equal duration (δ=1ms) and magnitude but opposite sign were used as flow-encoding gradients with amplitude ranging from 0.178 to 0.889 T/m dependent on shear rate-induced velocity, which were separated by a displacement observation time of Δ = 11 ms. The lateral direction (x) “read” gradients imaged the velocity with a 30 mm field of view using 512 points (58.6 μm resolution) across the diameter of the flow cell providing directly the velocity as a function of gap distance v(x).

Steady-state experiments used 16 scans (repetitions) of gradient flow-encoding steps over a total acquisition time of 1 min and 12 s, with the velocity measured representing an average velocity over this time. Unsteady-state (startup) experiments used 2 scans for a faster acquisition and acquired 75 independent velocity profiles averaged over 8.11 s for a total duration of approximately 10 min and 12 s. This transient measurement minimizes the velocity averaging time but lowers the acquired voltage signal-to-noise ratio. Velocimetry experiments were performed with shear and without shear for each shear rate to provide a no-flow control. This control allows for the removal of phase shifts from background magnetization during data analysis. All results shown represent the difference in the phase between flow and no-flow velocity profiles. The velocity-induced phase shifts map $\pm V_{\text{max}}$ to ±π in phase. Velocities larger than the velocity maximum with phase $\phi_{\text{max}}=\pi +\delta$ result in a phase wrap to -π+δ, and standard-phase unwrapping methods are used [39]. This ensures that imperfections from magnetic gradient fields do not affect the result.

## 3 Results and discussion

### 3.1 Rheometry

The viscosity and shear stress behavior of the NCC suspensions shown in Figure 1 is in agreement with published data [14,15]. The 3% w/v sample exhibits isotropic fluid behavior with a near-constant viscosity at low shear rate, $\dot{\gamma }$, and linear increase in shear stress. At 5% w/v, the classic 3-region behavior of nematic chiral liquid crystals [40,41] is evident with shear thinning at low $\dot{\gamma }$ up to ~1/s, followed by a slight plateau from 1 to 10/s and stronger shear thinning at high $\dot{\gamma }$ greater than 10/s [14,15]. The shear stress exhibits a plateau and slight decrease in the region from 1 to 10/s, which is consistent with shear banding and flow instability phenomenon [40,41]. When the concentration reaches 7% w/v, a near-constant shear-thinning behavior of viscosity, which is consistent with a gel-like phase, is initiated, while the shear stress shows a slight plateau in the 1–10/s region. By 12% w/v, the viscosity exhibits a constant shear-thinning behavior over the entire $\dot{\gamma }$ range with near-constant shear stress.

### 3.2 Rheo-NMR velocimetry

**Figure 4:**
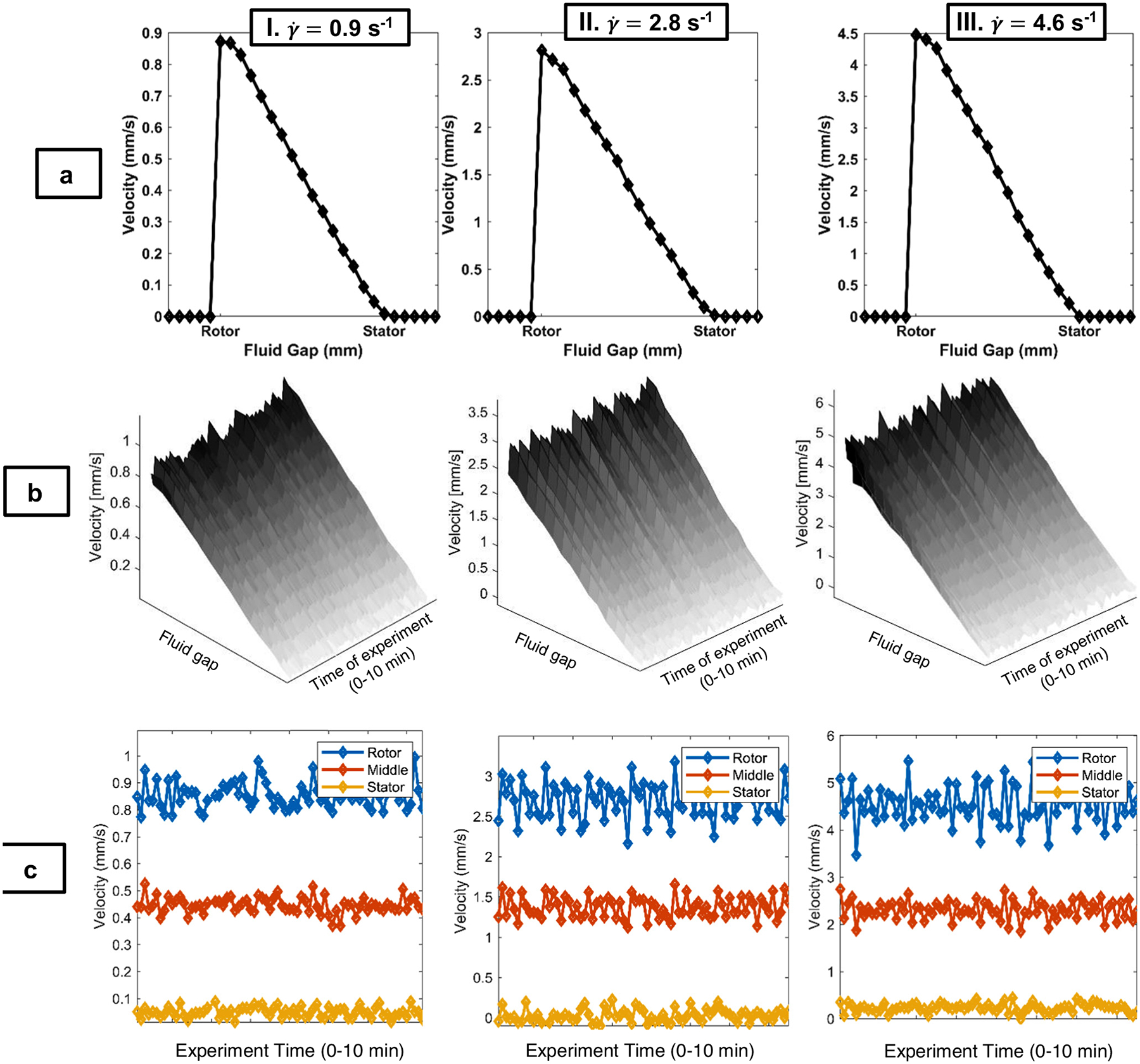
Rows a and b, columns I–III. 1D NMR velocimetry experiments for NCC2, 3% w/v suspension. Rows (a) steady-state, 1D velocity profiles, with 16 scans and total time of acquisition of 1 min and 12 s, and a pixel resolution of approximately 65 μm; (b) surface plots for 1D velocity profiles during an unsteady-state experiment with 75 experiments averaged over 2 scans and total time of acquisition of approximately 10 min and 12 s; (c) velocity profiles from the same unsteady-state experiment as (b), but displayed at three discrete positions across the 1 mm sample Couette cell fluid gap (rotor, middle, and stator) for all time points, which demonstrate the velocity changes across the experiment time and the fluid gap. Columns I: shear rate of $\dot{\gamma }=0.9/s$; II: shear rate of $\dot{\gamma }=2.8/s$; III: shear rate of $\dot{\gamma }=4.6/s$. The data present nearly Newtonian behavior for this concentration. Rotor and stator data points are approximately 58.6 μm from the physical walls.

The Rheo-NMR velocimetry results provide insight into the spatial and time dependence of the shear-induced motion. The lowest concentration (3% w/v) exhibits nearly Newtonian behavior in both start-up and steady-state experiments, depicted by a near-linear flow profile with no slip at the stator and less than 3% at the rotor (Figure 4a and b, Table 1). The linear velocity distribution reflects the near-isotropic fluid behavior exhibited in the rheology at the $\dot{\gamma }=0.9,2.8$, and 4.6 s^−1^ examined. The viscosity data indicate a slight shear-thinning behavior; however, for the narrow gap flow cell geometry, the deviation from linearity of the velocity distribution is small even for Ostwald de Waele power law indices of 0.5<n<1.0 [38]. In Figure 4c, the time-dependent velocities at the stator, gap center, and rotor are shown. The fluctuations in velocity are consistent with the noise of the stepper motor, as indicated by the Newtonian calibration silicone oil. Mean, μ=⟨v⟩, and standard deviation of velocity, $\sigma ={\left\langle v^{\prime 2}\right\rangle }^{1/2}$, are shown in Table 2 for all three gap positions and shear rates. The flow cell-induced velocity fluctuations as measured by the standard deviation increase with $\dot{\gamma }$ and decrease from rotor to stator with decreasing velocity across the gap.

**Figure 5:**
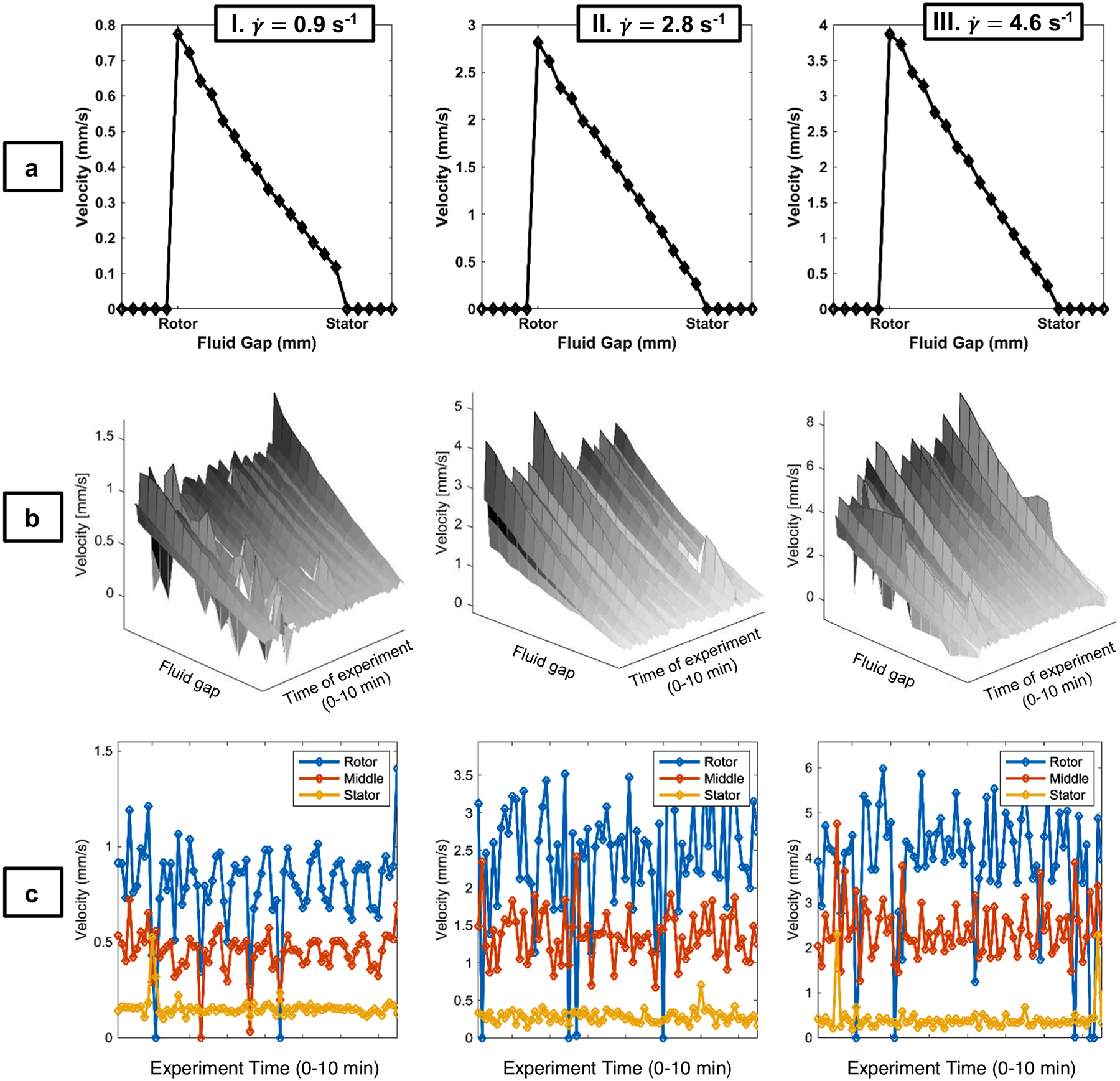
Rows a and b, columns I–III. 1D NMR velocimetry experiments for NCC3, 5% w/v suspension, a liquid crystal. Rows (a) steady-state, 1D velocity profiles, with 16 scans and total time of acquisition of 1 min, 12 s, and a pixel resolution of approximately 65 μm; (b) surface plots for 1D velocity profiles during an unsteady-state experiment with 75 experiments averaged over 2 scans and total time of acquisition of approximately 10 min and 12 s, which demonstrate velocity fluctuations; (c) velocity profiles from the same unsteady-state experiment as (b), but displayed at three discrete positions across the 1 mm sample Couette cell fluid gap (rotor, middle, and stator) for all time points, which demonstrate the velocity changes across the experiment time and the fluid gap; significant velocity fluctuations exist for this concentration. Columns I: shear rate of $\dot{\gamma }=0.9/s$; II: shear rate of $\dot{\gamma }=2.8/s$; III: shear rate of $\dot{\gamma }=4.6/s$. Rotor and stator data points are approximately 58.6 μm from the physical walls.

At 5% w/v for the chiral nematic phase, the steady-state velocity distributions (Figure 5a) remain linear, which is consistent with a shear-thinning fluid flow. The suspension begins to exhibit a significant slip at the stator and rotor, with the stator slip decreasing with increasing shear rate. The rotor slip as a percentage of the Newtonian no-slip velocity of silicone oil, defined as $\left(1-\frac{v_{\text{NCC}}}{v_{\text{SiOil}}}\right)\cdot 100\%$, provides a measure of the slip by comparing the velocity of the NCC sample at the pixel closest to the rotor compared to that of the no-slip silicone oil, which indicates the actual rotor velocity. Rotor slip values for 5% w/v (Table 1) are large at $\dot{\gamma }=0.9$ and 4.6/s, but not 2.8/s. The transient velocity distributions (Figure 5b) indicate significant spatial variations with shearing time for all $\dot{\gamma }$. In Figure 5c, the time-dependent velocity across the gap indicates significant fluctuations greater than for the Newtonian standard. The largest fluctuations in Figure 5b and c increase in frequency during the shearing time with increasing $\dot{\gamma }$. The standard deviations show a large increase in σ across the gap from stator to rotor, and we will return to a detailed discussion of this large fluctuation behavior in the context of liquid crystal flow instabilities below [39,42,43].

**Figure 6:**
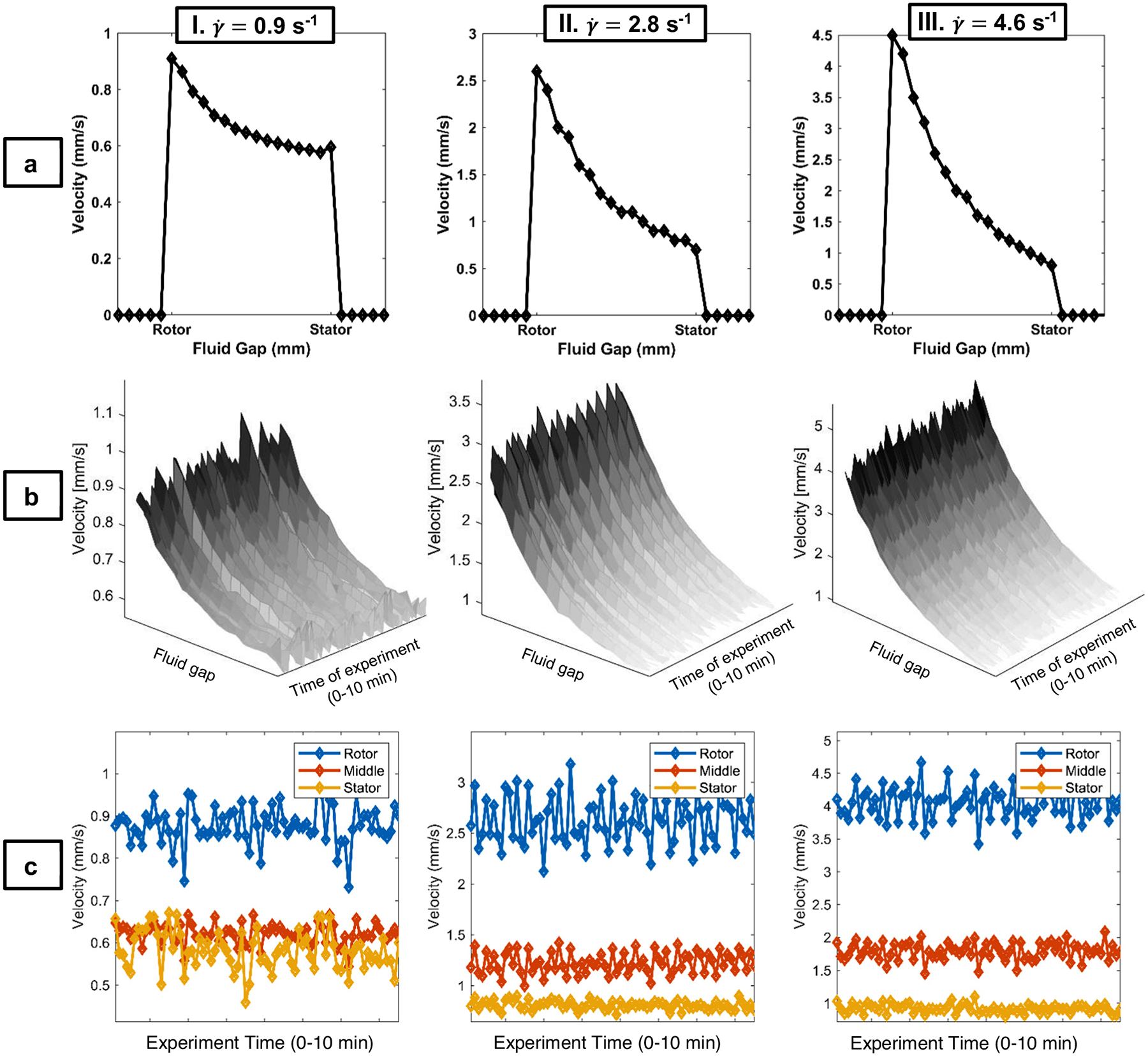
Rows a and b, Columns I–III. 1D NMR velocimetry experiments for NCC1, 7% w/v suspension, a gel. Rows (a) steady-state, 1D velocity profiles, with 16 scans and total time of acquisition of 1 min, 12 s, and a pixel resolution of approximately 65 μm; (b) surface plots for 1D velocity profiles during an unsteady-state experiment with 75 experiments averaged over 2 scans and total time of acquisition of approximately 10 min and 12 s; (c) velocity profiles from the same unsteady-state experiment as (b), but displayed at three discrete positions across the 1 mm sample Couette cell fluid gap (rotor, middle, and stator) for all time points, which demonstrate velocity changes across the experiment time and the fluid gap. Columns I: shear rate of $\dot{\gamma }=0.9/s$; II: shear rate of $\dot{\gamma }=2.8/s$; III: shear rate of $\dot{\gamma }=4.6/s$. The data present wall-slip and yielding behavior representative of a yield-stress gel. Rotor and stator data points are approximately 58.6 μm from the physical walls.

Increasing the NCC concentration to 7% w/v into the gel phase generates strong spatial dependence of the steady-state velocity distributions (Figure 6a). There is a significant stator slip decreasing with increasing $\dot{\gamma }$ and rotor slip decreasing from 10% of Newtonian fluid velocity at $\dot{\gamma }=0.9/s$ to 2% of Newtonian, which is similar to the 3% w/v sample at $\dot{\gamma }=4.6/s$. The decrease in slip at the highest $\dot{\gamma }$ indicates the microstructural disruption of the gel phase by the higher shear rate. Coupled effects of apparent wall slip and shear localization in shear banding and yield stress materials is a well-established phenomenon, which occurs in particulate dispersions [44]. Apparent wall slip in particle dispersions occurs in a thin layer of the suspending fluid [45]. The data indicate that apparent slip occurs in a single pixel, meaning the slip-layer thickness is much less than the 58.6 μm pixel resolution. The steady-state velocity distributions indicate shear banding or shear localization [22–29]. Coussot and co-workers have defined shear localization as a progressive continuous decrease in $\dot{\gamma }$ across a gap in contrast to a shear rate discontinuity for shear banding [25]. The measured steady-state velocity distributions (Figure 6a) have a continuous variation of $\dot{\gamma }$ but can be characterized by a region of high shear rate, ${\dot{\gamma }}_{H}$, near the rotor and constant low shear rate, ${\dot{\gamma }}_{L}$, near the stator (Table 1). At applied shear rate $\dot{\gamma }=0.9/s$, the ${\dot{\gamma }}_{H}$ is lower than the applied shear due to the 10% wall slip and ${\dot{\gamma }}_{L}$ occupies the majority of the fluid gap. The behavior at $\dot{\gamma }=2.8/s$ with smaller rotor slip demonstrates an increase in ${\dot{\gamma }}_{H}$ over the applied shear rate and a smaller gap ${\dot{\gamma }}_{L}$ region, indicating a greater disruption of the microstructure with the shear overcoming the particle negative surface charge repulsions. By applied $\dot{\gamma }=4.6/s,\;{\dot{\gamma }}_{H}$ is larger than the applied shear and occupies over half the gap (Table 3) and the low shear region is undergoing significant shear. The transient velocity distributions (Figure 6b and c) exhibit no large fluctuations in contrast to the 5% w/v sample despite a slight indication of a small plateau in the rheology data (Figure 1), and the standard deviation is similar to the Newtonian calibration fluid.

**Figure 7:**
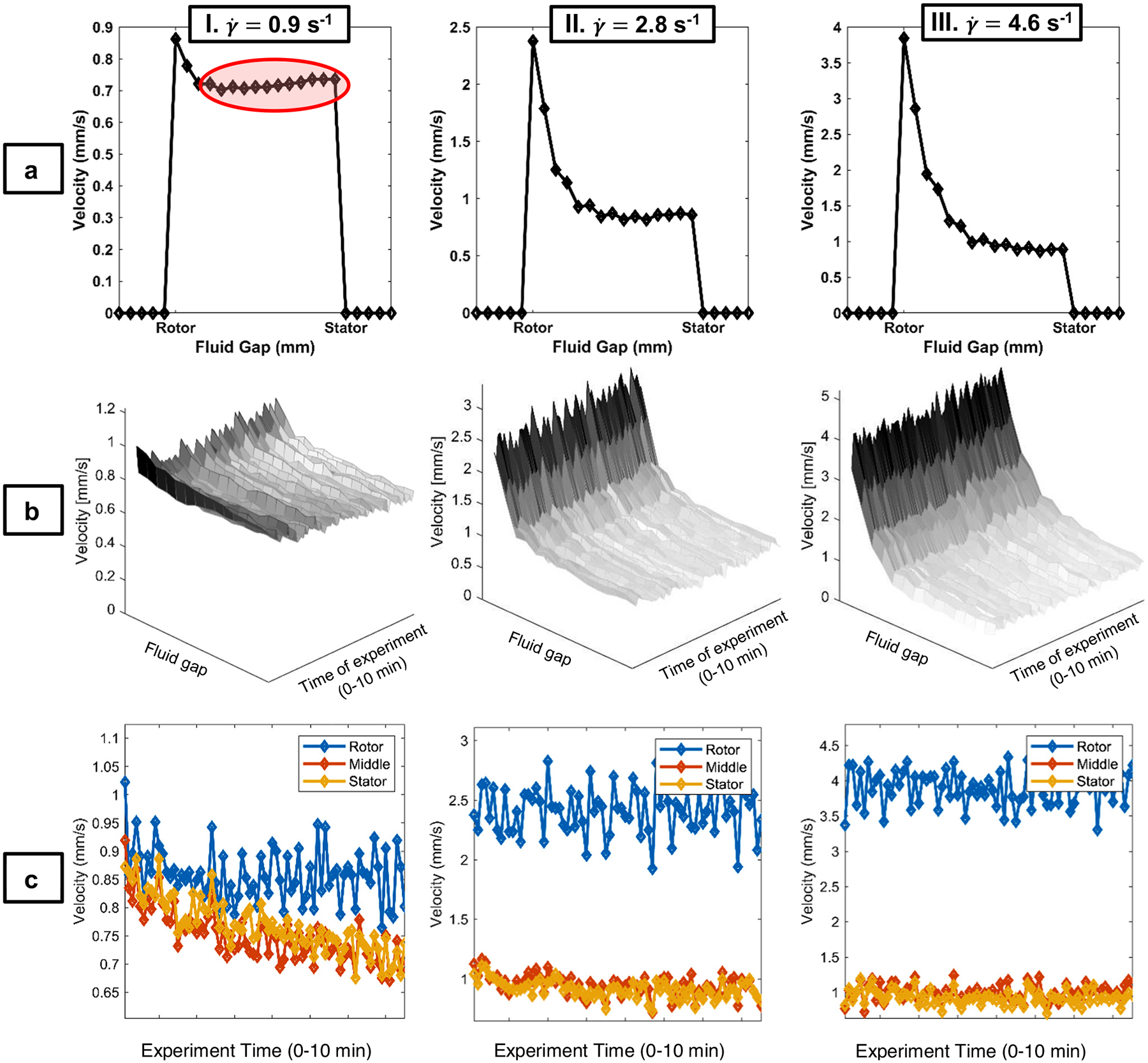
Rows a and b, Columns I-III. 1D NMR velocimetry experiments for NCC4, 12% w/v suspension, a very thick gel. Rows (a) steady-state, 1D velocity profiles, with 16 scans and total time of acquisition of 1 min, 12 s, and a pixel resolution of approximately 65 μm; (b) surface plots for 1D velocity profiles during an unsteady-state experiment with 75 experiments averaged over 2 scans and total time of acquisition of approximately 10 min and 12 s; (c) velocity profiles from the same unsteady-state experiment as (b), but displayed at three discrete positions across the 1 mm sample Couette cell fluid gap (rotor, middle, and stator) for all time points, which demonstrate velocity changes across the experiment time and the fluid gap. Columns I: shear rate of $\dot{\gamma }=0.9/s$; II: shear rate of $\dot{\gamma }=2.8/s$; III: shear rate of $\dot{\gamma }=4.6/s$. The data present wall-slip, shear-banding, and yielding representative of a thick yield-stress gel. The red shaded region indicates a region of solid-body-like rotation. Rotor and stator data points are approximately 58.6 μm from the physical walls.

The 12% w/v gel exhibits yield stress behavior. The slip at the rotor (Figure 7a) is comparable to 5% w/v, and at the stator, it is largest of all samples for all $\dot{\gamma }$. The steady-state velocity distributions (Figure 7a) exhibit an increasing region of shearing into the gap with increasing $\dot{\gamma }$. Of note is the increasing velocity with increasing distance across the gap (Table 3) in the no-shear region at $\dot{\gamma }=0.9$ and 2.8/s (Figure 7a I and II), which indicates the solid body rotation [36,38]. At $\dot{\gamma }=4.6/s$ (Figure 7a III), the shear rate in the near solid-like region is positive with ${\dot{\gamma }}_{L}=0.26/s$, indicating a disruption of the solid microstructure, which is present in the $\dot{\gamma }=0.9$ and 2.8/s applied shear rates as evidenced by the $\dot{\gamma }$ sign changes, where in Table 1 we define $\dot{\gamma }$ relative to the gap so that decreasing velocity across the gap is listed as positive $\dot{\gamma }$ and increasing velocity as negative $\dot{\gamma }$. The transient data for $\dot{\gamma }=0.9$ and 2.8/s (Figure 7b I and II and 7c I and II) show a longer timescale transient not present at lower concentrations or at $\dot{\gamma }=4.6/s$. This manifests as the clear progression of the velocity spatial distributions, for $\dot{\gamma }=0.9/s$ (Figure 7b I) to larger slip at both rotor and stator and in the time-dependent data (Figure 7c I) as a drift to lower velocities over the 10 min shearing times. These effects are also present in the solid body flow region at $\dot{\gamma }=2.8/s$ (Figure 7b II and 7c II). The temporal velocity fluctuation amplitudes as measured by the standard deviation are of order of the Newtonian calibration, indicating that the gel structure damps fluctuations relative to the 5% w/v sample.

### 3.3 Large-velocity fluctuations

The large fluctuations in transient velocity near the rotor generate large-velocity values greater than the rotor speed that (Figure 5c) are highly reproducible in the 5% w/v sample, which exhibits nematic liquid crystal-type rheology. Large fluctuations in velocity have been predicted for nematic liquid crystals in the simulations of Kupferman et al. using a long-range elastic correlation [43], for Ericksen numbers $\text{Er}=\frac{\eta {\dot{\gamma }}_{\text{eff}}}{\frac{K}{d^{2}}}\sim 400$ and Deborah number $\text{De}=\frac{\dot{\gamma }}{D_{R}}\sim 1$. Er is the ratio of viscous stress $\eta {\dot{\gamma }}_{\text{eff}}$, with η a characteristic Leslie viscosity ${\dot{\gamma }}_{\text{eff}}=v/d$, to the Frank elastic stress, where $K\sim \frac{u}{a}$ is the Frank constant proportional to the nematic interaction energy u and a the nematic length scale [40]. De is the ratio of the experimental rate of shear $\dot{\gamma }$ to the rate of relaxation of the nematic director $\underline{n}$, which is $1/D_{R}$, with $D_{R}$ being the nematic rotational diffusivity [40]. The nematic length scale a can be either the molecular or particle length scale or a supermolecular scale of microdomains of oriented nematics, which for the NCC particles is a~10μm, while the dimensionless interaction potential of the order of the Doi rigid rod model is u~10, and the rotational diffusivity is $D_{R}\sim 10^{2}/S$ [40,43,46]. For the NCC particles in this work and the d=1 mm flow gap, the data in Figure 5 are in the De ~ 10^−2^ and Er ~ 10^3^ regime for which tumbling of the particles occurs [40,42,43]. In the simulations of Kupferman et al. at De ~ 1 and Er ~ 400, intense vortex structures are formed due to large elastic energy release from the destruction of defect lines generated by rod tumbling, which result in large positive and negative velocities exceeding the wall velocity [43]. The frequency and amplitude of the large-velocity fluctuations (Figure 5c) scales with $\dot{\gamma }$, increasing with increasing $\dot{\gamma }$. In addition, chiral nematic liquid crystal mesophases are known to exhibit positional, orientational, and coupled positional–orientational slip at various length scales [40,42]. These modes contribute to the dynamic flow instabilities exhibited as velocity fluctuations in the velocimetry data. Ongoing research aims to determine the impact of each of these modes by applying PGSE NMR methods to characterize the length and timescale of these fluctuations as has been done in turbulent and granular flows [39,47].

Velocimetry methods have not previously been applied to study the rheological flows of NCC suspensions [13]. Novel Rheo-NMR velocimetry data with spatial and temporal resolution indicate that NCC suspensions undergo strongly varying flow deviations from linear in a Couette cell, which correlates with subtle variations in steady bulk rheology and includes wall-slip, shear banding, and yielding. The data indicate large-velocity fluctuations in the liquid crystal-phase suspension (5% w/v), seen for the first time in NCCs, due to particle director orientation tumbling and flow instability not clearly discernible in the bulk rheology. The results provide the details of the mesoscale velocity distributions on the order of 10’s μm, which allows the integration of the observed wall-slip and shear banding phenomena to model NCC rheology. This data is also relevant to the design of processing flow conditions where stress inhomogeneity can cause significant material microstructure change over scales of the order of 10’s μm that alter material properties. The data also clearly demonstrate the utility of using magnetic resonance velocimetry to study a range of processing flows of NCC.

## Figures and Tables

**Table 1:** Rotor slip percentage and localized shear rates at rotor $\left({\dot{\gamma }}_{H}\right)$ and stator $\left({\dot{\gamma }}_{L}\right)$

Shear rate [/s]	Variable	3% w/v	5% w/v	7% w/v	12% w/v
0.9	Rotor slip %	3.07	15.63	−1.10	4.17
	${\dot{\gamma }}_{H}$			0.82	0.60
	${\dot{\gamma }}_{L}$			0.11	(−0.07)
2.8	Rotor slip %	−0.44	−0.44	7.04	15.08
	${\dot{\gamma }}_{H}$			4.01	5.32
	${\dot{\gamma }}_{L}$			0.81	(−0.012)
4.6	Rotor slip %	2.69	15.89	2.12	16.40
	${\dot{\gamma }}_{H}$			5.31	2.95
	${\dot{\gamma }}_{L}$			1.63	0.26

**Table 2:** Mean (μ) and standard deviation (σ) of velocity at three gap positions for transient data

Shear rate [/s]	Position	3% w/v		5% w/v		7% w/v		12% w/v		10k cSt Si Oil	
		μ[mm/s]	σ[mm/s]	μ[mm/s]	σ[mm/s]	μ[mm/s]	σ[mm/s]	μ[mm/s]	σ[mm/s]	μ[mm/s]	σ[mm/s]
0.9	Rotor	0.86	0.05	0.80	0.22	0.88	0.05	0.86	0.05	0.88	0.04
	Middle	0.45	0.03	0.41	0.12	0.63	0.03	0.75	0.05	0.46	0.02
	Stator	0.05	0.02	0.16	0.05	0.58	0.05	0.77	0.05	0.04	0.01
2.8	Rotor	2.71	0.23	2.51	0.82	2.63	0.24	2.42	0.20	2.75	0.23
	Middle	1.37	0.13	1.23	0.31	1.23	0.10	0.95	0.09	1.44	0.12
	Stator	0.03	0.08	0.29	0.08	0.81	0.04	0.91	0.08	0.13	0.03
4.6	Rotor	4.55	0.40	4.17	1.34	4.06	0.25	3.91	0.26	4.62	0.32
	Middle	2.31	0.20	2.10	0.59	1.79	0.13	1.06	0.12	2.42	0.17
	Stator	0.23	0.09	0.35	0.12	0.92	0.06	0.93	0.10	0.20	0.07

**Table 3:** Gap positions (mm), where ${\dot{\gamma }}_{H}$ and ${\dot{\gamma }}_{L}$ intersect or solid body rotation initiates for gel concentrations

Shear rate [/s]		7% w/v		12% w/v
0.9	Intersection	0.39	Solid body	0.19
2.8	Intersection	0.41	Solid body	0.31
4.6	Intersection	0.53	Intersection	0.49

## Data Availability

All data generated or analyzed during this study are included in this published article.

## Abbreviations

NCC: nanocrystalline cellulose
NMR: nuclear magnetic resonance
PGSE: pulsed gradient spin echo

## References

[1] Rongpipi S, Ye D, Gomez ED, Gomez EW. Progress and opportunities in the characterization of cellulose – an important regulator of cell wall growth and mechanics. Front Plant Sci. 2019;9:1894. 30881371 10.3389/fpls.2018.01894 PMC6405478

[2] McNamara JT, Morgan JL, Zimmer J. A molecular description of cellulose biosynthesis. Annu Rev Biochem. 2015;84:895–921. 26034894 10.1146/annurev-biochem-060614-033930 PMC4710354

[3] Bhatia SK, Jagtap SS, Bedekar AA, Bhatia RK, Patel AK, Pant D, Recent developments in pretreatment technologies on lignocellulosic biomass: Effect of key parameters, technological improvements, and challenges. Bioresour Technol. 2020;300:122724. 31926792 10.1016/j.biortech.2019.122724

[4] Carolin CF, Kamalesh T, Kumar PS, Hemavathy RV, Rangasamy G. A critical review on sustainable cellulose materials and its multifaceted applications. Ind Crop Prod. 2023;203:117221.

[5] Delidovich I, Hausoul PJC, Deng L, Pfützenreuter R, Rose M, Palkovits R. Alternative monomers based on lignocellulose and their use for polymer production. Chem Rev. 2016;116(3):1540–99. 26523853 10.1021/acs.chemrev.5b00354

[6] Raj T, Chandrasekhar K, Naresh Kumar A, Kim S-H. Lignocellulosic biomass as renewable feedstock for biodegradable and recyclable plastics production: A sustainable approach. Renew Sustain Energy Rev. 2022;158:112130.

[7] Trache D, Tarchoun AF, Derradji M, Hamidon TS, Masruchin N, Brosse N, Nanocellulose: From fundamentals to advanced applications. Front Chem. 2020;8:392. 32435633 10.3389/fchem.2020.00392 PMC7218176

[8] Ong X-R, Chen AX, Li N, Yang YY, Luo HK. Nanocellulose: Recent advances toward biomedical applications. Small Sci. 2023;3(2):2200076. 40213493 10.1002/smsc.202200076 PMC11935994

[9] Ghilan A, Nicu R, Ciolacu DE, Ciolacu F. Insight into the latest medical applications of nanocellulose. Materials (Basel). 2023;16(12):15–27. 10.3390/ma16124447 PMC10303580 37374630

[10] Shojaeiarani J, Bajwa DS, Chanda S. Cellulose nanocrystal based composites: A review. Compos Part C: Open Access. 2021;5:100164.

[11] George J, Sabapathi SN. Cellulose nanocrystals: synthesis, functional properties, and applications. Nanotechnol Sci Appl. 2015;8:45–54. 26604715 10.2147/NSA.S64386 PMC4639556

[12] Singh S, Bhardwaj S, Tiwari P, Dev K, Ghosh K, Maji PK. Recent advances in cellulose nanocrystals-based sensors: a review. Mater Adv. 2024;5(7):2622–54.

[13] Kadar R, Spirk S, Nypelo T. Cellulose nanocrystal liquid crystal phases: Progress and challenges in characterization using rheology coupled to optics, scattering, and spectroscopy. ACS Nano. 2021;15(5):7931–45. 33756078 10.1021/acsnano.0c09829 PMC8158857

[14] Shafiei-Sabet S, Hamad WY, Hatzikiriakos SG. Rheology of nanocrystalline cellulose aqueous suspensions. Langmuir. 2012;28(49):17124–33. 23146090 10.1021/la303380v

[15] Liao J, Pham KA, Breedveld V. Rheological characterization and modeling of cellulose nanocrystal and TEMPO-oxidized cellulose nanofibril suspensions. Cellulose. 2020;27(7):3741–57.

[16] Cainglet HE, Tanner J, Nasiri N, Browne C, Garnier G, Batchelor W. Rapid cellulose nanomaterial characterisation by rheology. Cellulose. 2023;30(8):4971–82.

[17] Gray DG. Order and gelation of cellulose nanocrystal suspensions: an overview of some issues. Philos Trans R Soc A: Math, Phys Eng Sci. 2018;376(2112):20170038. 10.1098/rsta.2017.0038 PMC5746553 29277736

[18] Stroobants A, Lekkerkerker HNW, Odijk T. Effect of electrostatic interaction on the liquid crystal phase transition in solutions of rodlike polyelectrolytes. Macromolecules. 1986;19(8):2232–8.

[19] Xu Y, Atrens AD, Stokes JR. Rheology and microstructure of aqueous suspensions of nanocrystalline cellulose rods. J Colloid Interface Sci. 2017;496:130–40. 28214623 10.1016/j.jcis.2017.02.020

[20] Zakani B, Grecov D. Effect of ultrasonic treatment on yield stress of highly concentrated cellulose nano-crystalline (CNC) aqueous suspensions. Carbohydr Polym. 2022;291:119651. 35698354 10.1016/j.carbpol.2022.119651

[21] Echeverria C, Almeida PL, Feio G, Figueirinhas JL, Rey AD, Godinho MH. Rheo-NMR study of water-based cellulose liquid crystal system at high shear rates. Polymer. 2015;65:18–25.

[22] Callaghan PT. Rheo-NMR and velocity imaging. Curr Opin Colloid Interface Sci. 2006;11(1):13–8.

[23] Rogers SA, Vlassopoulos D, Callaghan PT. Aging, yielding, and shear banding in soft colloidal glasses. Phys Rev Lett. 2008;100(12):128304. 18517918 10.1103/PhysRevLett.100.128304

[24] Holmes WM, Lopez-Gonzalez MR, Callaghan PT. Fluctuations in shear-banded flow seen by NMR velocimetry. Europhys Lett. 2003;64(2):274–80.

[25] Ovarlez G, Rodts S, Chateau X, Coussot P. Phenomenology and physical origin of shear localization and shear banding in complex fluids. Rheol Acta. 2009;48(8):831–44.

[26] Britton MM, Callaghan PT. Two-phase shear band structures at uniform stress. Phys Rev Lett. 1997;78(26):4930–3.

[27] Coussot P Progress in rheology and hydrodynamics allowed by NMR or MRI techniques. Exp Fluids. 2020;61(9):6–8.

[28] Al-kaby RN, Codd SL, Seymour JD, Brown JR. Characterization of velocity fluctuations and the transition from transient to steady state shear banding with and without pre-shear in a wormlike micelle solution under shear startup by Rheo-NMR. Appl Rheol. 2020;30(1):1–13.

[29] Brox TI, Douglass B, Galvosas P, Brown JR. Observations of the influence of Taylor-Couette geometry on the onset of shear-banding in surfactant wormlike micelles. J Rheol. 2016;60(5):973–82.

[30] Davies CJ, Sederman AJ, Pipe CJ, McKinley GH, Gladden LF, Johns ML. Rapid measurement of transient velocity evolution using GERVAIS. J Magn Reson. 2010;202(1):93–101. 19897390 10.1016/j.jmr.2009.10.004

[31] Siebert H, Grabowski DA, Schmidt C. Rheo-NMR study of a non-flow-aligning side-chain liquid crystal polymer in nematic solution. Rheol Acta. 1997;36(6):618–27.

[32] Geng Y, Almeida PL, Feio GM, Figueirinhas JL, Godinho MH. Water-based cellulose liquid crystal system investigated by Rheo-NMR. Macromolecules. 2013;46(11):4296–302.

[33] Chanda S, Bajwa DS, Holt GA, Stark N, Bajwa SG, Quadir M. Silane compatibilization to improve the dispersion, thermal and mechanical properties of cellulose nanocrystals in poly (ethylene oxide). Nanocomposites. 2021;7(1):87–96.

[34] Beck-Candanedo S, Roman M, Gray DG. Effect of reaction conditions on the properties and behavior of wood cellulose nanocrystal suspensions. Biomacromolecules. 2005;6(2):1048–54. 15762677 10.1021/bm049300p

[35] Casado U, Mucci VL, Aranguren MI. Cellulose nanocrystals suspensions: Liquid crystal anisotropy, rheology and films iridescence. Carbohydr Polym. 2021;261:117848. 33766344 10.1016/j.carbpol.2021.117848

[36] Callaghan PT. Rheo NMR and shear banding. Rheol Acta. 2008;47(3):243–55.

[37] Al-kaby RN, Jayaratne JS, Brox TI, Codd SL, Seymour JD, Brown JR. Rheo-NMR of transient and steady state shear banding under shear startup. J Rheol. 2018;62(5):1125–34.

[38] Jayaratne JS, Codd SL, Al-Kaby RN, Maley J, Brox TI, Galvosas P, Large amplitude oscillatory shear rheo-NMR velocimetry. Phys Fluids. 2023;35(9):093105.

[39] Callaghan PT. Translational dynamics and magnetic resonance: principles of pulsed gradient spin echo NMR. Oxford, England: Oxford University Press; 2011.

[40] Larson RG. The structure and rheology of complex fluids. Oxford, England: Oxford University Press; 1999.

[41] Onogi S, Asada T. Rheology and rheo-optics of polymer liquid crystals. In: Astarita G, Marrucci G, Nicolais L, editors. Rheology: Volume 1: Principles. Boston, MA: Springer US; 1980. p. 127–47.

[42] Rey AD, Denn MM. Dynamical phenomena in liquid-crystalline materials. Annu Rev Fluid Mech. 2002;34:233–66.

[43] Kupferman R, Kawaguchi MN, Denn MM. Emergence of structure in a model of liquid crystalline polymers with elastic coupling. J Non-Newton Fluid. 2000;91(2–3):255–71.

[44] Cloître M, Bonnecaze RT. A review on wall slip in high solid dispersions. Rheol Acta. 2017;56:283–305.

[45] Buscall R Letter to the Editor: Wall slip in dispersion rheometry. J Rheol. 2010;54(6):1177–83.

[46] Van Rie J, Schütz C, Gençer A, Lombardo S, Gasser U, Kumar S, Anisotropic diffusion and phase behavior of cellulose nanocrystal suspensions. Langmuir. 2019;35(6):2289–302. 30672300 10.1021/acs.langmuir.8b03792

[47] Seymour JD, Caprihan A, Altobelli SA, Fukushima E. Pulsed gradient spin echo nuclear magnetic resonance imaging of diffusion in granular flow. Phys Rev Lett. 2000;84(2):266–9. 11015887 10.1103/PhysRevLett.84.266

